# The relationship between midwife-led group-based versus conventional antenatal care and mode of birth: a matched cohort study

**DOI:** 10.1186/s12884-016-1216-1

**Published:** 2017-01-19

**Authors:** Lauren Kearney, Mary Kynn, Alison Craswell, Rachel Reed

**Affiliations:** 1 University of the Sunshine Coast, Locked Bag 4, Maroochydore DC, 4558 Queensland Australia; 2 Women and Families Service Group, Sunshine Coast Hospital and Health Service, Maroochydore DC, Queensland Australia

**Keywords:** Midwifery, Antenatal education, Pregnancy, Prenatal care, Caesarean section, Caesarean birth

## Abstract

**Background:**

Midwife facilitated, group models of antenatal care have emerged as an alternative to conventional care both within Australia and internationally. Group antenatal care can be offered in a number of different ways, however usually constitutes a series of sessions co-ordinated by a midwife combining physical assessment, antenatal education and peer support in a group setting. Midwife-led group antenatal care is viewed positively by expectant mothers, with no associated adverse outcomes identified in the published literature for women or their babies when compared with conventional care. Evidence of an improvement in outcomes is limited. The aim of this study was to compare mode of birth (any vaginal birth with caesarean birth) between pregnant women accessing midwife-led group antenatal care and conventional individual antenatal care, in Queensland, Australia.

**Methods:**

This was a retrospective matched cohort study, set within a collaborative antenatal clinic between the local university and regional public health service in Queensland, Australia. Midwife-led group antenatal care (*n* = 110) participants were compared with controls enrolled in conventional antenatal care (*n* = 330). Groups were matched by parity, maternal age and gestation to form comparable groups, selecting a homogeneous sample with respect to confounding variables likely to affect outcomes.

**Results:**

There was no evidence that group care resulted in a greater number of caesarean births. The largest increase in the odds of caesarean birth was associated with a previous caesarean birth (*p* < 0.001), no previous birth (compared with previous vaginal birth) (*p* < 0.003), and conventional antenatal care (*p* < 0.073). The secondary outcomes (breastfeeding and infant birth weight) which were examined between the matched cohorts were comparable between groups.

**Conclusions:**

There is no evidence arising from this study that there was a significant difference in mode of birth (caesarean or vaginal) between group and conventional care. Group care was associated with a lower risk of caesarean birth after controlling for previous births, with the highest chance for a vaginal birth being a woman who has had a previous vaginal birth and was in group care. Conversely, the highest risk of caesarean birth was for women who have had a previous caesarean birth and conventional care.

## Background

Most women in Australia receive antenatal care routinely during their pregnancy [1]. Conventional antenatal care in Australia usually involves women seeing numerous health care professionals, individually in a clinic setting, during the course of their pregnancy. These care providers include hospital midwives and doctors, General Practitioners and community-based services. Child birth education or classes are also offered as an adjunct for this health care, on an opt-in basis. The recently developed Australian National Clinical Practice Guidelines recommend a minimum antenatal schedule of ten visits up to, and including a 40 week (term) appointment [2, 3]. Midwife facilitated, group models of antenatal care (hereafter referred to as midwife-led group antenatal care) have emerged as an alternative to conventional care both within Australia [4] and internationally [5]. Midwife-led group antenatal care can be offered in a number of different ways, however usually constitutes a series of sessions co-ordinated by a midwife combining physical assessment, antenatal education and peer support in a group setting [6].

Midwife-led group antenatal care has been studied most widely in North America where the CenteringPregnancy model of group antenatal care was developed [5]. The most recent systematic review found midwife-led group antenatal care to be positively viewed by expectant mothers, with no associated adverse outcomes for women or their babies when compared with conventional care [7]. Preterm birth rates were not affected by group or conventional antenatal care [7, 8] however, reduced rates have been reported for midwife led continuity of care models [9]. An earlier integrative review conducted in 2011 had reported lower rates of preterm birth associated midwife-led group antenatal care [10], however, this was not sustained in the most recent systematic review of the evidence. There were only a small number of studies from which to draw this conclusion, with 42% of the women in the meta-analysis from one study. This evidence supports what other authors have reported: that there is a need to conduct further research to determine whether or not benefit to women and babies is associated with midwife-led group antenatal care [7].

Qualitative studies on group antenatal care have reported high maternal satisfaction, peer support and the value of shared experience associated with group antenatal care [11], especially models that include continuity of care [12]. Women accessing midwife-led group antenatal care models demonstrate healthier pregnancies [10, 13] and improved antenatal care attendance amongst disadvantaged groups [14] compared to conventional antenatal care. Breastfeeding rates have been shown to be higher following midwife-led group antenatal care [13, 15].

In the context of the positive outcomes associated with midwife-led group antenatal care, it follows that providing it as one option for women is appropriate. However, in the shrinking fiscal environment of healthcare, new models of care require evaluation to demonstrate models are evidence-based and at least *as good as* or better than what is already offered. “Rising healthcare costs and limited provider availability have intensified the search for evidence-based, cost-effective prenatal care that maximizes the outcomes of the mother and child” [16]. However, the relationship between midwife-led group antenatal care and mode of birth (caesarean or vaginal) has not been established [7]. Models of midwifery care which have been shown to affect mode of birth reside within continuity of midwifery care throughout the perinatal continuum, for low-risk pregnant women [17]. Yet, this same effect on mode of birth has not been demonstrated in all-risk continuity of care models [18, 19].

Given the steadily rising caesarean birth rates both within Australia and internationally [1, 20, 21], innovative models of antenatal care may afford one opportunity to combat this trend, given the potentially high complications associated with caesarean birth. Sandall and colleagues [22] argue that “Whilst it is difficult to exclusively categorise maternity models of care due to the influence of generic policies and guidelines, it is assumed that the underpinning philosophy of a midwifery model of care is on normality and the natural ability of women to experience birth with minimum or no routine intervention” [22]. Therefore this study aimed to compare outcomes (primarily proportion of caesarean births) between a midwifery-led group based antenatal care service (known as ‘Expecting and Connecting’) with conventional antenatal care in a regional area of Queensland, Australia.

## Methods

### Study design and setting

A matched cohort study was conducted in a regional setting in south-east Queensland, Australia. This is the second phase of a larger study evaluating the ‘Expecting and Connecting’ service and the earlier phase (qualitative findings) are reported elsewhere [11]. All pregnant women who attended a ‘booking-in’ appointment within the local, public health care service were given an opportunity to opt-in to the ‘Expecting and Connecting’ group pregnancy care service, run at the campus of a local university or attend another of the conventional service models available at the local public hospital. There were a range of options for conventional antenatal care including combinations of Medical practitioner (public hospital clinic), Midwifery practitioner (public hospital clinic); or, Medical practitioner–General Practitioner (shared care).

The ‘Expecting and Connecting’ group pregnancy care service (ECGPC) commenced as a collaborative partnership between a regional health service and local university in 2013. This service facilitates midwife-led group antenatal care for up to 12 women for seven sessions, six antenatal sessions and one postnatal. Lasting around two hours each, the sessions include a comprehensive antenatal assessment and facilitated educational discussion. Topics include preparation for labour and birth, adjusting to parenting and pregnancy care and allow for interaction and discussion, rather than a didactic, expert approach. Operating from the local university, sessions are facilitated collaboratively by one health service midwife, and one university midwife. Women commence sessions at around 18–20 weeks gestation. The model facilitates continuity of midwife during the antenatal period, but does not extend into the labour and birthing period.

Pregnant women who opted in to the ECGPC (intervention), were matched with a carefully selected comparison cohort experiencing conventional care. The control group accessed a range of options for antenatal care and for analysis, any combination of public prenatal care other than attendance at ECGPS was termed conventional antenatal care as this is the range of options available to all women attending as a public patient at the local hospital (study setting).

The null hypothesis was that the proportion of women experiencing any vaginal birth would be the same in the ‘Expecting and Connecting’ group pregnancy care group as in the conventional antenatal care group.

### Sampling

The required sample size was approximated using a background CB rate of 0.25, confidence level of 95%, and 80% power using published sample size tables for logistic regression [23]. Based on these tables a final sample size between 339 and 549 would be sufficient to detect an odds ratio for risk of CB between groups of 1.3–1.4 or greater.

Women who had received antenatal care through ECGPC service were identified and considered as cases for inclusion in the study. A total of just over 300 women had accessed ECGPC service since its inception in early 2013. To allow time for establishment of the new service, and to collect data from the most recent representation of clients through the service, a sample period from January, 2014 to Dec 2014, was selected. A total of 119 cohort cases were identified with nine case records unable to be matched to the Queensland Perinatal Data Collection (QPDC), as the mothers birthed in 2015. This resulted in 110 cases being included in the intervention group. The control group comprised a matched sample accessed via QPDC. Three controls per case were selected based on the following shared attributes: Term (Preterm <37 weeks, Term > =37 weeks), Parity (Nulliparous/Multiparous), Mother Age (5 years Groups), Singleton births and they gave birth at the local public hospital. The matching resulted in the selection of 330 controls. These controls were selected from 1 June 2012 through to 31 December 2012. This process of matching characteristics between the intervention and control group has been reported in other Australian health care research studies with a ratio of 1:3 [24, 25]. All linked data were cross-checked with controls on all matching criteria. Data corresponded at 100% accuracy for maternal age, gestational age at birth and parity. Therefore a total sample of 440 was achieved. It should be noted that matching on pre-term birth removes the possibility of detecting any difference in this variable. However this decision was made to remove any bias in the primary outcome of mode of birth (CB or VB) as policies for pre-term birth may result in a higher incidence of caesarean birth [8].

### Data collection

For the control group all data items were extracted from QPDC. QPDC data were not included for the case file as the majority of the case data supplied included mothers that gave birth in 2014 or later. At the time of this analysis 2014 QPDC data was not finalised. The data for the cases were accessed by clinical record audit.

The primary outcome was mode of birth, categorised as any vaginal birth (VB) or caesarean birth (CB). Secondary outcomes included: low birth weight (<2500 g at term); and, breastfeeding on discharge from hospital. Potential explanatory factors were prenatal care, pharmaceutical pain relief, mode of previous births, maternal age and gestational age.

### Data analysis

All data were entered into the statistical analysis program SPSS (version 22, IBM Corp, Armonk, New York, USA). Descriptive statistics were calculated for demographic, primary and secondary outcome variables for each group.

Descriptive statistics were calculated for the two cohorts. T-tests and chi-squared tests were conducted to confirm groups were approximately equivalent after matching.

Logistic regression was used to calculate adjusted odds ratios (aOR) and 95% confidence intervals (CI) where the dependent variable was mode of birth (CB or any VB). Antenatal care, pharmaceutical pain relief in labour, mode of previous births, maternal age and gestational age were tested as explanatory variables.

## Results

Demographic characteristics of the groups were very similar (Table 1–Characteristics of groups). Although mothers were matched in 5 year age groups there was a small but significant difference in the mean age of the mothers, with the group care being about 1 year older on average. There were no other significant differences in the characteristics of the two groups, including use of pain relief and birth weight.

**Table 1:** Characteristics of Groups

Variables	Group Care	Standard care	Total
	(*n* = 110) No. (%)	(*n* = 330) No. (%)	(*n = ###*) No. (%)
Maternal Age^a,*^ [mean (SD)]	29 (5.3)	28 (5.2)	28 (5.3)
Gestational Age^a^			
Term (> = 37 weeks)	107 (97.3)	321 (97.3)	428 (97.3)
Preterm (<37 weeks)	3 (2.7)	9 (2.7)	12 (2.7)
Parity^a^			
Primigravida	80 (72.7)	237 (71.8)	320 (72.7)
Multigravida	30 (27.3)	93 (28.2)	120 (27.3)
Previous Births			
No previous birth	80 (72.7)	237 (71.8)	317 (72.0)
Previous SVB	28 (25.4)	73 (22.1)	92 (20.9)
Previous LSCS	2 (1.8)	20 (6.1)	22 (5.0)
Infant birth weight^b^			
>4500 g	2 (1.8)	7 (2.1)	9 (2.0)
4001–4500 g	17 (15.5)	32 (9.7)	49 (11.1)
3501–4000 g	34 (30.9)	122 (37.0)	156 (35.5)
3001–3500 g	39 (35.5)	130 (39.4)	169 (38.4)
2501–3000 g	14 (12.7)	29 (8.8)	43 (9.8)
2001–2500 g	4 (3.6)	2 (0.6)	12 (2.7)

^a^Matching variable

^b^Some categories with low cell counts were merged with neighbouring categories for a valid test

^*^Difference between groups significant (*p* < 0.05)

Results from the logistic regression analysis are presented in table two comparing conventional antenatal care with group regarding mode of birth (Table 2).

**Table 2:** Associations between caesarean section (CS) and risk factors in a multivariate logistic regression model

Factors	CS		VB		Adjusted OR (95% CI)	*P*-value
	No	%	No	%		
Ante-natal care						
Group care	19	*17.8*	91	*27.3*	1	
Standard care	88	*82.2*	242	*72.7*	1.7 (0.95, 3.04)	0.073
Use of nitrous						
Yes	33	*30.8*	206	*61.9*	1	
No	74	*69.2*	127	*38.1*	4.0 (2.45, 6.51)	0.000
Previous births						
Previous VB	13	*12.1*	88	*26.4*	1	
Previous CS	15	*14.0*	7	*2.1*	14.8 (4.76, 46.05)	0.000
No previous birth	79	*73.8*	238	*71.5*	2.7 (1.42, 5.28)	0.003

There was no evidence that group care resulted in a greater number of Caesarean births, and some evidence that group care reduced the odds of a Caesarean birth. The use of epidural or spinal analgesia cannot be used in the logistic regression model for mode of birth as the rate for caesarean birth is 100% and causal pathway is unclear (for example, a planned CB will involve spinal analgesia where the planning of the caesarean birth comes first, however, in a prolonged or difficult labour an epidural or spinal analgesia may be used as pain relief and a CB may be subsequently advised). The use of opioids was not significantly associated with mode of birth and removed from the analysis.

Maternal Age and gestational age were not significant, however the overall low rate of preterm births suggests that to detect a significant effect would require a much larger sample size.

Breastfeeding rates on discharge were comparable between groups (88.1% v 87.9%; *p* < 0.3). The usual length of hospital stay is 2 days for women birthing vaginally within the study site. Breastfeeding initiation rates were not collected as they are known to be above 96% within the study site and a very large sample would be required to detect a difference.

The original data did not determine whether CB was elective or emergency, however the confirmed use of nitrous oxide as pain relief indicated that those cases reflect a woman who has laboured prior to the birth mode of CB, rather than a non-labour elective CB. Therefore, the increased odds of a CB for women who did not use nitrous was likely a statistical artefact.

## Discussion

There is no evidence arising from this study that there is a difference in mode of birth (caesarean or vaginal) rates between group and conventional care. While a difference was found, it was not significant to *p* < 0.05 that group care is associated with a lower risk of caesarean birth after controlling for previous births (none/caesarean birth/vaginal birth). In this study, the highest chance for a vaginal birth was a woman who has had a previous vaginal birth and was in group care. Conversely, the highest risk of caesarean birth was for women who have had a previous caesarean birth and conventional care.

Ruiz-Mirazo and colleagues also found a slightly decreased risk of caesarean birth in their systematic review and meta-analysis of midwife-led group antenatal care when compared with individual care [15]. There remains however, large variation in the ways in which group-based antenatal care can be provided and the way in which it is facilitated. The findings from this study can be applied to the unique context of the ‘Expecting and Connecting’ model. Beckmann and colleagues [19] conducted a large retrospective cohort study examining the effect Midwifery Group Practice had on mode of birth, also in Queensland Australia. Their study found no difference on mode of birth (primary outcome unassisted vaginal birth) between Midwifery Group Practice and conventional care. Yet the Midwifery Group Practice (MGP) model is quite different from ‘Expecting and Connecting’, as the women are cared for in a continuity of care model (in MGP), with the group dynamics and approach substantially different from the structure and focus of ‘Expecting and Connecting’ or other facilitative group approaches, such as CenteringPregnancy. Group facilitation skills of midwives may be a variable to consider when comparing the way the antenatal group care is provided [26]. The depth of information and shared experience is also a valuable component of midwife-led group antenatal care. Jenkins et al. [27] found that informational continuity is essential for women during pregnancy and childbirth as they can undergo multiple interactions with many differing healthcare providers. Continuity of relationship (relational continuity) is seen as particularly important to pregnant women in minimising the number of healthcare professionals they interact with during pregnancy.

The recent systematic review comparing group with conventional antenatal care on pregnancy and birth outcomes [7], also found no difference on perinatal outcomes such as prematurity or mode of birth, however report high levels of maternal satisfaction and no demonstration of harm. Unlike many MGP or continuity of care models, ‘Expecting and Connecting’ does not specifically target ‘low risk’ women, rather all pregnant women are informed of the service and invited to participate should they choose to.

Furthermore, the ECGPC model was very popular amongst primigravid women (72% of the case cohort). Nulliparity has been associated with an increased risk for a CB without a clear medical indication [28], and nulliparous women have been identified as the group with the most modifiable factors present which can be manipulated to reduce CB, and as such have been listed as a core maternity quality indicator in the United States of America [29], Despite this, there has been a significant increase in women birthing via CB in their first pregnancy in Australia over the past 20 years, and this has led to an ongoing increase in repeat CB [1, 21]. This study found that the strongest predictor of a CB was a previous CB, and that the strongest indicator of a VB was a previous VB. Therefore, it has been argued that strategies aimed at reducing CB rates should focus on promoting and supporting VB for primigravid women [30].

The secondary outcomes which were examined between the matched cohorts were also comparable between groups. Previous studies have demonstrated an increase in breastfeeding initiation from group-care [15]. Our study did not demonstrate a significant difference in exclusive breastfeeding initiation between the matched cohorts (88.1% v 87.9%; *p* < 0.3), however considering the very high rates of breastfeeding on discharge from the study health service hospital (87% fully breast feeding, 9% combination of breast milk and infant formula, 4% infant formula [31]), much larger numbers would be required to detect difference. It would be perhaps more important to ascertain the potential effect midwife-led group antenatal care may have on breastfeeding duration, especially in the context of the peer-support generated through the service, and is an area for further research.

### Limitations

Women who attended the intervention in this study (‘Expecting and Connecting’) self-selected into this group, in preference to other models of conventional antenatal care offered through the local public hospital and health service. Other studies have reported that women choosing MGP approaches tend to be older [19], however this study has aimed to control for maternal age, and other key confounding factors known to influence mode of birth.

The matched cases from the QPDC were drawn from late 2012 data, as this was the most current validated data at the time of linkage. The case data were extracted from the local birth registration database from 2014. No significant changes had occurred in the health service or modifications made to conventional antenatal care during this time, so should have little or no impact on the data integrity.

Controlling for emergency or elective caesarean birth and longer follow-up period for breastfeeding duration would be important to consider for future research. Further, the number of women who had previously accessed ‘Expecting and Connecting’ determined the limited sample size (case group) for this study and as such could only have the power to detect large differences in caesarean birth rates, therefore future research with larger cohorts of pregnant women would be valuable. Similarly, instrumental and spontaneous vaginal births were grouped as ‘any vaginal birth’ and it is important to acknowledge that for some women an instrumental vaginal birth may be more traumatic than a caesarean birth. Therefore it would be useful to power future studies to differentiate between elective and emergency CB and spontaneous and instrumental VB. The findings of this study are specific to the context within which the study was undertaken and should be interpreted as such.

## Conclusion

Considering the known adverse effects of caesarean birth, especially on subsequent pregnancies and births [32–34], innovative and supportive models of antenatal care which mitigate this risk, especially for nulliparous women are essential. The high levels of reported satisfaction [35] and no increase in adverse outcomes sees midwife-led group antenatal care as a viable option for antenatal care. There are mixed findings within the literature about the impact of various models of maternity care on mode of birth outcome. However, specific to the ‘Expecting and Connecting’ collaborative approach, a non-significant reduction in odds of caesarean birth was demonstrated and further experimental research with larger cohorts examining this in the Australian context would be useful.

## Abbreviations

CB: Caesarean birthl
MGP: Midwifery Group Practice
QPDC: Queensland Perinatal Data Collection
VB: Vaginal birth (incorporating for the purpose of this study spontaneous and instrumental vaginal birth)
